# Transcranial Magnetic Stimulation Alleviates Levodopa-Induced Dyskinesia in Parkinson's Disease and the Related Mechanisms: A Mini-Review

**DOI:** 10.3389/fneur.2021.758345

**Published:** 2021-11-08

**Authors:** Yi Wu, Xue-bing Cao, Wei-qi Zeng, Heng Zhai, Xiao-qian Zhang, Xiao-man Yang, Chi Cheng, Jia-ling Wang, Xiao-mei Yang, Yan Xu

**Affiliations:** Department of Neurology, Union Hospital, Tongji Medical College, Huazhong University of Science and Technology, Wuhan, China

**Keywords:** transcranial magnetic stimulation, Parkinson's disease, dyskinesia, mechanism, treatment

## Abstract

After long-term use of levodopa, Parkinson's patients almost inevitably develop dyskinesia, a kind of drug side effect manifesting as uncontrollable choreic movements and dystonia, which could be crippling yet have limited therapeutic options. Transcranial magnetic stimulation is the most widely studied non-invasive neuromodulation technology to treat levodopa-induced dyskinesia. Many studies have shown that transcranial magnetic stimulation has beneficial effects on levodopa-induced dyskinesia and is patient-tolerable, barely with reported adverse effects. Changes in brain connectivity, neuroplasticity, neurotransmitter, neurorestoration, and blood flow modulation could play crucial roles in the efficacy of transcranial magnetic stimulation for levodopa-induced dyskinesia. The appearance of new modes and application for emerging targets are possible solutions for transcranial magnetic stimulation to achieve sustained efficacy. Since the sample size in all available studies is small, more randomized double-blind controlled studies are needed to elucidate the specific treatment mechanisms and optimize treatment parameters.

## Introduction

Parkinson's disease (PD) is a neurodegenerative disease characterized by the degeneration of substantia nigra dopaminergic neurons. Levodopa is the mainstay drug choice in the clinical management of PD. However, long-term levodopa supplements convert Parkinson's patients from akinetic state to hyperkinetic state, namely levodopa-induced dyskinesia (LID), with its severity ranging from mild and barely noticeable to severely disabled. After 4–6 years of levodopa administration, the occurrence rate of dyskinesia is 40%, while after 15 years, the occurrence rates can be up to 94% (1).

Modifying dopaminergic therapy to provide more continuous dopaminergic stimulation is helpful for the management of LID. Apart from dopaminergic drugs, amantadine is currently regarded as the most effective drug for LID treatment (2). Although the efficacy of amantadine has been proved to be long-lasting and remarkable, its use might induce/exacerbate unacceptable hallucinations and also is contraindicated in patients with end-stage renal disease (3). Discontinuation of this drug is even associated with a significant risk of worsening dyskinesias (3). These undesirable effects limited the long-term use of amantadine. Several other chemicals targeting adenosine, adrenergic, glutamatergic, and serotonergic receptors have significantly decreased dyskinesias in animal models but not in parkinsonian patients (3). For patients refractory to medical management, neurosurgical approaches are also the procedure of choice. Among them, deep brain stimulation has been widely studied and recommended as one priority procedure for LID patients who need surgery. However, deep brain stimulation costs much, needs regular follow-up appointments over several years, and bears a danger of possible adverse effects after electrode placement (4). Surgical ablation of the globus pallidus has been reported to have remarkable efficacy in treating contralateral dyskinetic symptoms, while its efficacy is more petite than bilateral subthalamic nucleus deep brain stimulation in one case of a randomized controlled trial (4). A new minimally invasive approach using magnetic resonance-guided focused ultrasound to ablate globus pallidus has only been shown to improve dyskinesia in a case report (4).

In contrast, another non-invasive procedure, transcranial magnetic stimulation (TMS), has been applied to treat LID since 2005 (5) and has shown some benefits to a certain extent in several studies. TMS might be a promising neuromodulation skill to improve LID. The purpose of the present review is to discuss the main points of TMS in the management of LID and related mechanisms to allow for a better understanding of its potential uses.

## An Outline for Studies Concerning Transcranial Magnetic Stimulation in the Management of LID

### Studies Utilizing Repetitive Transcranial Magnetic Stimulation

A pilot study subjected 8 PD patients with LID to 1 day of 15-min, low-frequency (1 Hz) rTMS (LF-rTMS) over the supplementary motor area (SMA) during apomorphine infusion (5). Mean (average of two raters) dyskinesia was significantly lower immediately and 15 min after the LF-rTMS sessions but not 30 min afterward (5). Brusa et al. conducted the same LF-rTMS on 10 PD patients with LID over SMA after levodopa intake (6). Unlike the pilot study, besides 15-min, single-day stimulation, this study also observed the effect of repeated, 5-day stimulation (6). This study found that mean (average of two raters) dyskinesia were significantly lower 15 and 30 min but not 45 and 60 min after the single-day and 5-day LF-rTMS sessions (6). Also, both single- and multiple-session LF-rTMS increased dyskinesia onset latency to the same degree comparing with sham control and no rTMS condition (6).

A later study conducted a 10-day LF-rTMS protocol over the primary motor cortex (MC) for 6 PD patients with LID after levodopa intake (7). Peak (during peak ON) and mean (average of early ON, peak ON, and late ON) dyskinesia were significantly lower for up to 1 day but not 2 weeks, whereas cortical excitability remained no change for all these time points (7). Although this study did not conduct sham control, we still could make some preliminary conclusions from it. Firstly, alterations of cortical excitability might not be the only mechanism involving the efficiency of LF-rTMS since cortical excitability did not correlate with the observed improvements in LID in this study. Secondly, more prolonged stimulation after-effects could be attained by extending stimulation days appropriately, such as 10 days rather than only 5 days. Positive relations between longer-lasting reduction of LID and longer sessions could be affirmed if further studies could apply longer-session LF-rTMS, such as a 3-week or even 4-week course.

A sham-controlled study later applied 4 consecutive days of LF-rTMS on 10 PD patients with LID during levodopa intake (8). In this study, a single session per day was increased from the previous 15 to 32 min (8). One-day reduction of LID severity was observed (8). However, it was a pity that this study did not record when the efficacy of LF-rTMS disappeared. Comparing with the outcome from Wagle-Shukla et al. (7) this finding suggests prolonged after-effects might also be obtained by increasing daily stimulation duration besides stimulation days (8). Also, this study firstly found that the major effect of LF-rTMS on LID improvement was on dystonia subscores (8).

Sayin et al. performed 10 consecutive days (30 min daily) of LF-rTMS over SMA on 17 PD patients with LID during levodopa intake (9). The study replicated 1 day of alleviation for LID, but the efficacy disappeared up to 120 min on the second day (9). Since this is a parallel sham controlled study, discrepancies of baseline dyskinesia severity between two groups might bring bias to outcomes (9). All aforementioned studies showed LF-rTMS had beneficial effects on LID improvement.

However, a later study showed adverse outcomes both after single-session and 5-day-multiple-session LF-rTMS (10). This was the first study to use two separate coils on bilateral MC; such an unexpected outcome might result from an offset of bilateral stimulation (10). It is speculated that a positive ipsilateral effect could be counterbalanced by a subsequent contralateral LF-rTMS session influencing more distant areas because previous studies have shown LF-rTMS ability to induce changes in areas distant from the stimulated area (11).

Lohse et al. firstly applied LF-rTMS over the pre-supplementary motor area (pre-SMA) on 17 PD patients with LID before levodopa intake (12). They found LF-rTMS utilization could help improve LID symptoms transiently (12). This was also the sole study regarding the relationship between stimulation intensity of LF-rTMS and its clinical impact on LID. Stimulation intensity is documented as the percentage of maximum stimulator output (MSO). With MSO of LF-rTMS increasing up to 60%, Lohse et al. found a significant linear correlation between stimulation intensity and individual prolongation of the time to onset of dyskinesia after levodopa intake (12). They also found a similar trend between MSO and individual reduction in dyskinesia severity, but it did not reach statistical significance (12). Recently, Flamez et al. conducted single-session LF-rTMS (16 min daily) over pre-SMA on 17 PD patients with LID before levodopa intake but failed to replicate the therapeutic effect on LID (13).

**Figure 1:**
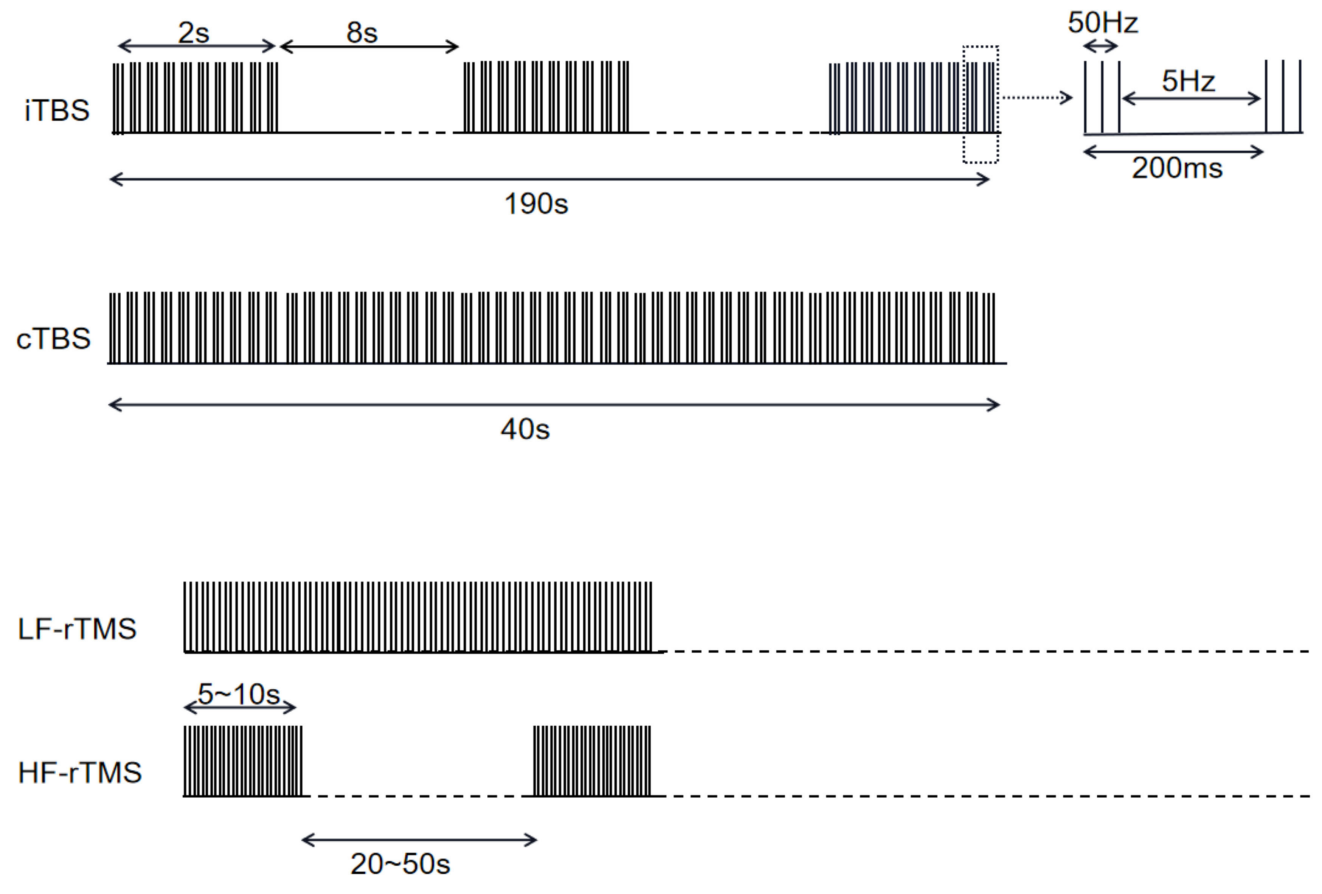
Overview of stimulation modes in studies mentioned in Tables 1, 2.

**Figure 2:**
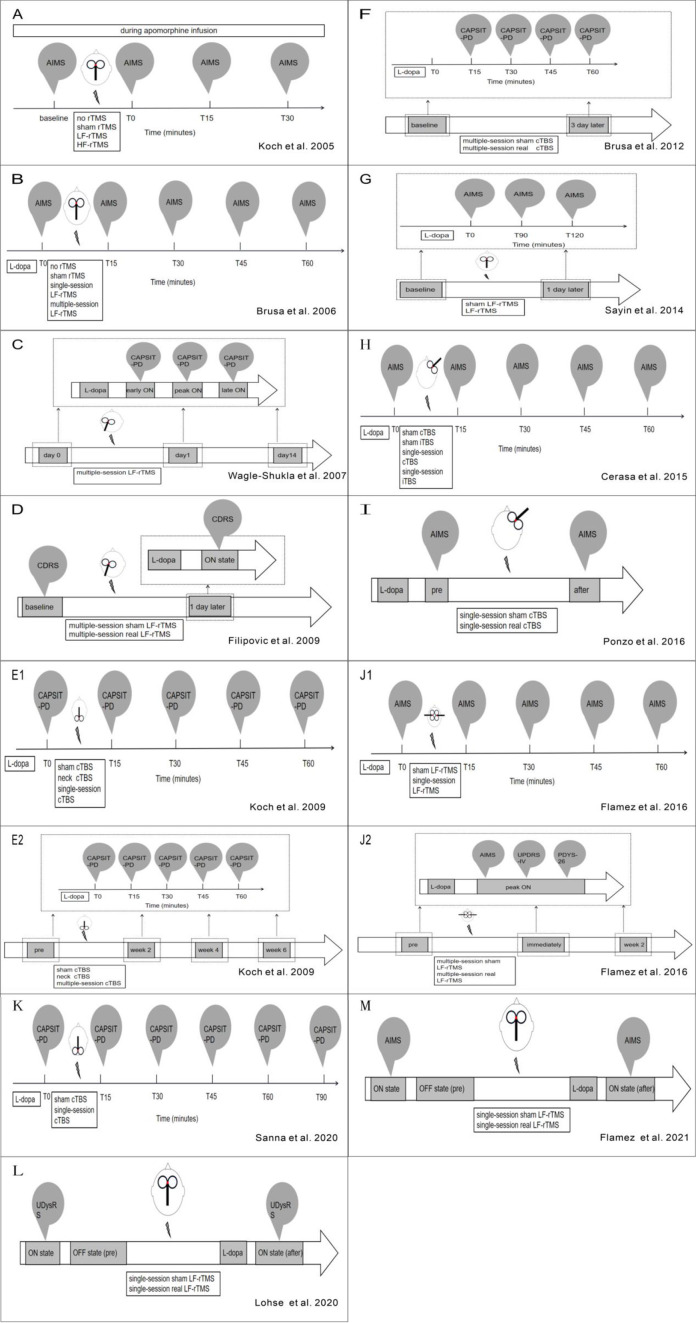
**(A–L)** Refers to overview of stimulation workflow in studies mentioned in Table 1.

Overall, most of these studies validated the short-term beneficial effect of LF-rTMS, but long-term therapeutic effects still needed to be explored. Among these studies, no adverse event was reported. Moreover, these beneficial effects are less likely to be induced by placebo effects. LF-rTMS seems to be a potential approach to treat LID. However, the conclusions from these studies are limited by the small sample sizes used. Also, differences in pharmacological status, dyskinesia assessment scales, and stimulation parameters (Table 1, Figures 1, 2) can confound outcomes of these LF-rTMS studies. Thus, once a mode of LF-rTMS with definite, reproducible, and sustained improvement on LID is established, LF-rTMS might be one of the most valuable approaches to alleviate LID in clinical settings.

**Table 1:** Overview of inhibitory TMS (LF rTMS/cTBS) for the treatment of LID in PD.

**Sample target**	**Coil design**	**TMS administration**	**Degree and scale of dyskinesia**	**Findings**	**References**
8	Bilateral 1 central coil	Single session (15 min* 1 day) of 1 Hz	Disabling	Improvement for	(5)
SMA	Sham controlled	LF-rTMS during apomorphine infusion	AIMS	15 min	
10	Bilateral 1 central coil	Single session (15 min* 1 day) of 1 Hz	Disabling	Improvement for	(6)
SMA	Sham controlled	LF-rTMS after levodopa intake	AIMS	30 min	
10	Bilateral 1 central coil	Multiple sessions (15 min* 5 days) of	Disabling	improvement for	
SMA	Sham controlled	1 Hz LF-rTMS after levodopa intake	AIMS	30 min	
6	Unilateral no coil type	Multiple sessions (15 min* 10 days)	Bothersome	Improvement for	(7)
MC	No sham	of 1 Hz LF-rTMS after levodopa intake	CAPSIT-PD	1 day	
10	Unilateral 1 central coil	Single session (40 s* 1 day) of cTBS	Disabling	Improvement for	(14)
Cerebellum	Sham controlled	After levodopa intake	CAPSIT-PD	45 min	
20	Bilateral 1 central coil	Multiple sessions (40 s* 10 days) of cTBS	Disabling	Improvement for	
Cerebellum	Sham controlled	After levodopa intake	CAPSIT-PD	4 weeks	
10	Unilateral no coil type	Multiple sessions (32 min* 4 days) of	Obvious	Improvement for	(8)
MC	Sham controlled	1 Hz LF-rTMS during levodopa intake	CDRS	1 day	
8	Bilateral 1 central coil	Multiple sessions (40 s* 5 days) of cTBS	Disabling	Improvement for	(15)
Cerebellum	Sham controlled	After levodopa intake	CAPSIT-PD	45 min	
17	Bilateral 1 central coil	Multiple sessions (30 min* 10 days) of	Disabling	Improvement for	(9)
SMA	Sham controlled	1 Hz LF-rTMS during levodopa intake	AIMS	1 day	
8	Unilateral no coil type	Single session (40 s* 1 day) of cTBS	Bothersome	Improvement for	(16)
IFC	Sham controlled	After levodopa intake	AIMS	30 min	
8	Unilateral no coil type	Single session (40 s* 1 day) of cTBS	Bothersome	No change	
MC	Sham controlled	After levodopa intake	AIMS		
9	Bilateral 2 separate coils	Single session (16 min* 1 day) of 1 Hz	Bothersome	No change	(10)
MC	Sham controlled	LF-rTMS during levodopa intake	AIMS,UPDRSIV,PDYS-26		
6	Bilateral 2 separate coils	Multiple sessions (16 min* 5 days) of 1 Hz	Bothersome	No change	
MC	Sham controlled	1 Hz LF-rTMS during levodopa intake	AIMS,UPDRSIV,PDYS-26		
10	1 central coil	Single session (40 s* 1 day) of cTBS	Disabling	Improvement for	(17)
IFC	Sham controlled	After levodopa intake	AIMS	No exact time	
11	1 central coil	Single session (40 s* 1 day) of cTBS	Bothersome	Improvement for	(18)
Cerebellum	Sham controlled	After levodopa intake	CAPSIT-PD	60 min	
17	1 central coil	Single session (30 min* 1 day) of 1 Hz	Obvious	Improvement for	(12)
preSMA	Sham controlled	LF-rTMS before levodopa intake	UDysRS	No exact time	
17	Unilateral 1 central coil	Single session (16 min* 1 day) of	No mention	No change	(13)
preSMA	Sham controlled	1 Hz LF-rTMS before levodopa intake	AIMS		

*PD, Parkinson's disease; LF rTMS, low-frequency repetitive transcranial magnetic stimulation; cTBS, continuous theta-burst stimulation; LID, levodopa-induced dyskinesia; SMA, supplementary motor area; MC, motor cortex; IFC, Inferior Frontal Cortex; AIMS, Abnormal Involuntary Movement Scale; mAIMS, Modified Abnormal Involuntary Movement Scale; CAPSIT-PD, Core Assessment Program for Surgical Interventional Therapies; LF-ADLS, Lang-Fahn Activities of Daily Living Scale; CDRS, Clinical Dyskinesia Rating Scale; PDYS-26, dyskinesia scale; VAS, Visual Analog Scale; UPDRS, Unified PD Rating Scale; UDysRS, Unified Dyskinesia Rating Scale*.

On the other hand, it was shown that all high frequency (5 and 10 Hz) rTMS (HF-rTMS) studies (Table 2, Figure 1) have no effect on LID.

**Table 2:** Overview of excitatory TMS (HF rTMS/iTBS) for the treatment of LID in PD.

**Sample target**	**Coil design**	**TMS administration**	**Degree and scale of dyskinesia**	**Findings**	**References**
8	Bilateral 1 central coil	Single session (15 min* 1 day) of 5 Hz	Disabling	No change	(5)
SMA	Sham controlled	HF-rTMS during apomorphine Infusion	AIMS		
4	Unilateral 1 central coil	Multiple sessions (no exact time* 5 days)	No mention	No change	(19)
DLPFC	Sham controlled	of 10 Hz HF-rTMS during levodopa intake	UPDRSIV		
4	Unilateral 1 central coil	Multiple sessions (no exact time* 5 days)	No mention	No change	
MC	Sham controlled	of 10 Hz HF-rTMS during levodopa intake	UPDRSIV		
8	Unilateral no coil type	Single session (40 s* 1 day) of iTBS	Bothersome	No change	(16)
IFC	Sham controlled	After levodopa intake	AIMS		
8	Unilateral no coil type	Single session (40 s* 1 day) of iTBS	Bothersome	No change	
MC	Cross-over sham controlled	After levodopa intake	AIMS		

*PD, Parkinson's disease; HF rTMS, high-frequency repetitive transcranial magnetic stimulation; iTBS, intermittent TBS; LID, levodopa-induced dyskinesia; SMA, supplementary motor area; MC, motor cortex; DLPFC, left dorsolateral prefrontal cortex; AIMS, Abnormal Involuntary Movement Scale; UPDRS, Unified PD Rating Scale*.

### Studies Utilizing Theta Burst Stimulation

Unlike rTMS, the protocol of TBS is comparatively more consistent among studies (Table 1, Figures 1, 2). For all five studies utilizing continuous TBS (cTBS), cTBS consists of three-pulse bursts at 50 Hz repeated every 200 ms for 40 s (20) and was administered after levodopa intake.

In Koch's study, they firstly applied single-session cTBS on 10 PD patients with LID over the cerebellum, and a 45-min reduction was observed (14). In this study, a 10-day course of cTBS was further conducted and induced persistent clinical beneficial effects up to 4 weeks (14). However, a later study applied a 5-day course of cTBS on 8 PD patients with LID over the cerebellum only reduced LID up to 45 min (15).

A study applied single-session cTBS over the inferior frontal cortex (IFC) and MC on 8 PD patients with LID, respectively (16). Stimulation over the right IFC induced improvement of LID only up to 30 min, while stimulation over MC did not exhibit any change (16). Although efficacy duration was not mentioned, Ponza et al. also observed the beneficial effect of cTBS on LID symptoms after single-session stimulation over the right IFC (17). A recent study targeting cerebellum also displayed 60-min alleviation for LID after cTBS stimulation (18).

Among these cTBS studies, two have mentioned specific stimulation intensity. In Koch's and Cerasa's studies (14, 16), 46.2 ± 8.5% MSO applied over the right IFC and cerebellum alleviated LID symptoms, while the same stimulation intensity over MC failed to improve LID symptoms. Since Cerasa et al. did not conduct further study to see whether higher stimulation intensity over MC would change the result or not, it could be early to deny the role of stimulation intensity for cTBS efficacy.

Like LF-rTMS, the short-term benefits of cTBS have been corroborated in several studies and are patient-tolerable. Although a remarkably longer after effect of cTBS than of LF-rTMS was exhibited only in one study, such prolonged effect did not replicate in other studies.

When it comes to intermittent theta-burst stimulation (iTBS) mode applied on IFC or MC (Table 2, Figure 1), no change has occurred to LID symptoms at both regions.

## An Outline for Stimulation Targets in TMS Protocols From the Studies Above

### Brain Regions in Motor Basal Ganglia Loop

MC is a crucial brain region involving in the development of LID. Alterations of potentials recorded from MC shed light on possible mechanisms underlying the benefits of LF-rTMS and cTBS for LID.

Short-interval intracortical inhibitions (SICI) and long-latency intracortical inhibition (LICI) reflect suppression of MC excitability (21, 22). In off therapy, SICI and LICI were decreased in PD patients with and without LID (23). Unlike PD patients without LID, administration of levodopa could not reverse decreased SICI and LICI in PD patients with LID (23). In off therapy, γ-Aminobutyric acid (GABAergic) agonist increased SICI in PD patients (24). Administration of GABAergic agonist could also alleviate LID (25). It is believed that SICI is likely to be mediated by GABA-A-ergic receptors (26) and LICI by GABA-B-ergic receptors (27, 28).

On the contrary, intracortical facilitation (ICF) and short-interval intracortical facilitation (SICF) reflect the facilitation of MC excitability (21, 29). Regardless of drug condition, ICF was found to decrease or remain normal in PD patients with LID (23, 30). Unlike ICF in dyskinetic patients, SICF kept increased in off and on the state (30). Such increase was positively correlated with the severity of LID (30). Increased SICF in LID patients could be alleviated by anti-glutamatergic drugs (30). Improvement of LID did not come with restoration of SICF (30), which suggests additional pathophysiological mechanisms might contribute to LID.

Findings of the two opposite types of potentials both indicated overexcitability of MC renders occurrence of LID. HF-rTMS (31) and iTBS (20) increases cortical excitability, whereas LF-rTMS (31, 32) and cTBS (20) decreases cortical excitability, which conforms to their opposite effects on LID symptoms. Apart from alterations of these potentials, dendritic spines in intratelencephalic-type corticostriatal neurons in MC became enlarged of rats with LID (33).

Although SMA did not show any structural modifification in PD patients with LID (34), neuroimaging has linked overactive SMA with the occurrence of LID (35, 36). Indeed, inhibitory LF-rTMS over SMA improved LID symptoms (5, 6, 9).

### Brain Regions in Associative and Limbic Basal Ganglia Loop

Voxel based morphometry reveals increased gray matter volume of the bilateral IFC in dyskinetic patients (34). Right IFC engages in suppressing an already initiated manual response (37). One study further revealed dyskinetic PD patients have a weaker inhibitory interaction between the right IFC and contralateral MC (17). This finding conforms with beneficial effects of inhibitory cTBS over right IFC on LID (17). Another study revealed that connectivity of the right IFC with the left MC was decreased in patients with LID (16). Nevertheless, inhibitory cTBS over right IFC improved LID symptoms in this study as well (16). Authors speculated that the increased communication between the right IFC and the putamen observed in this study in patients with LID might interfere with the motor inhibition network (16).

Task-based functional magnetic resonance imaging revealed activation of pre-SMA after intake of levodopa in LID patients (38). The pre-SMA has been implicated in both the suppression and initiation of movements (39). This might partly explain the contradictory outcomes of two LF-rTMS studies over pre-SMA on LID (12, 13).

Activation of the dorsolateral prefrontal cortex (DLPFC) was also observed in PD patients with LID (40). However, it was bewildering that HF-rTMS Stimulation of the left DLPFC induced a significant MC depression (19). Moreover, such MC depression did not reach a significant reduction of LID symptoms (19).

### Cerebellum

Increased metabolic activity in the dentate nucleus (15) and in the red nucleus (41) indicated cerebellar involvement in the development of LID. Further studies revealed cerebellar-cortical interaction in dyskinetic patients. After delivery of inhibitory cTBS over cerebellum, alleviated LID symptoms concurrently accompanied by enhancement of MC plasticity (42). Also resting-state functional connectivity was found to increase between cerebellum and left IFC: the greater the enhancement of cerebellar-IFC functional connectivity, the shorter was the latency of dyskinesia onset (43).

**Figure 3:**
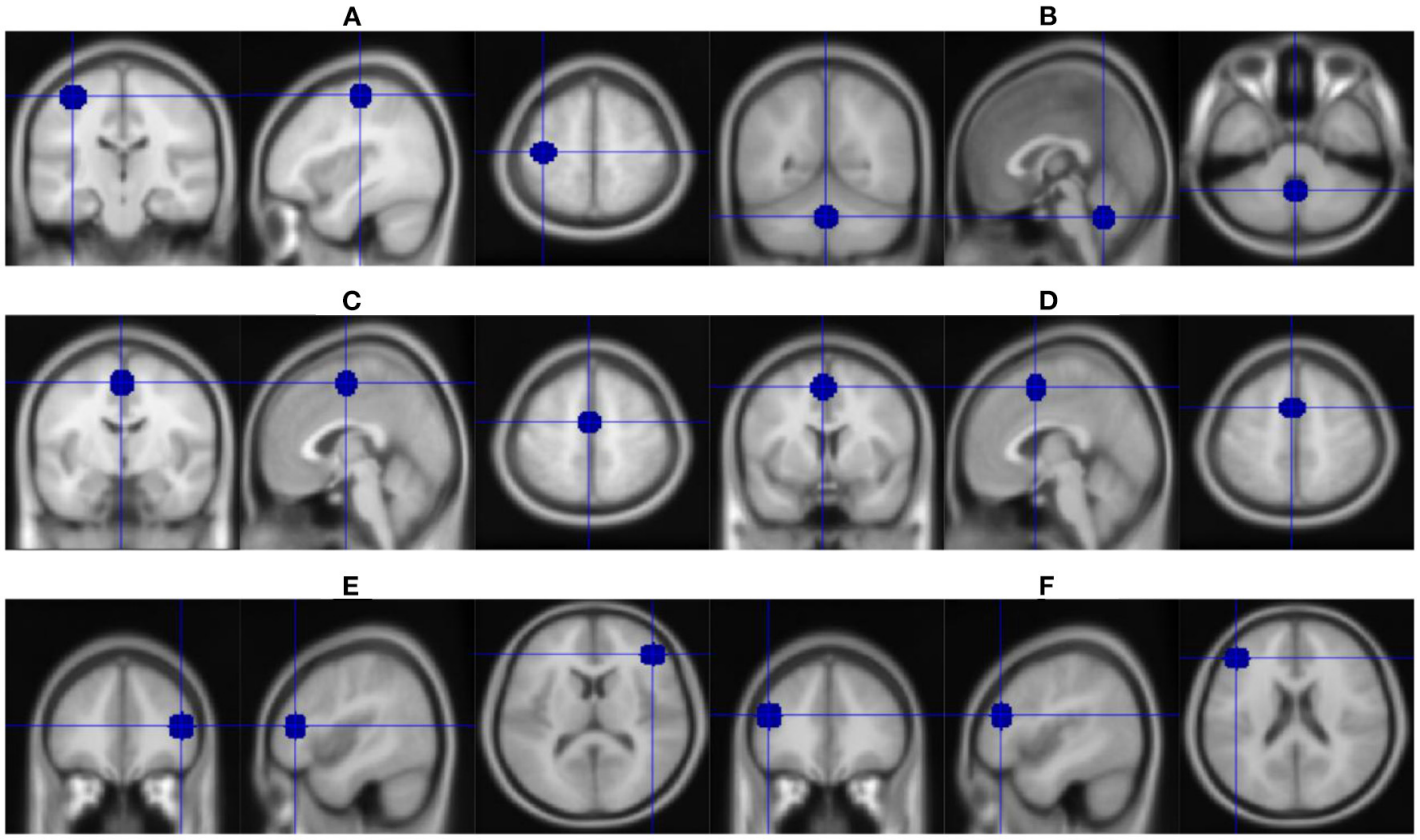
Overview of stimulation brain regions in studies mentioned in Tables 1, 2. **(A)** Left motor cortex. **(B)** Cerebellum. **(C)** Supplementary motor area. **(D)** Pre-supplementary motor area. **(E)** Right inferior frontal cortex. **(F)** Left dorsolateral prefrontal cortex.

Since many circuits take part in the occurrence of LID, identification of the critical brain region (Figure 3) involved in all LID mechanisms as the stimulation target or combination of different regions might prolong treatment efficacy.

## The Therapeutic Mechanism of TMS in the Clinical Management of LID

### Brain Connectivity

Electrophysiology and functional imaging are helpful to explore the role of brain connectivity in the occurrence of LID. Cross-frequency coupling (CFC) refers to a phenomenon that oscillations recorded by microelectrodes in various brain regions interact with each other (44). Such CFC is presented as a quantitative value to show inter-brain synchrony (44). It is revealed that CFC between MC and dorsolateral striatum was decreased in LID rats (44). After delivery of HF-rTMS, hippocampus-prefrontal CFC in patients with major depression was enhanced (45). How LF-rTMS and cTBS alleviate LID symptoms by the change of CFC remains unknown.

### Neuroplasticity

MC lacks long-term potentiation (LTP) -like synaptic plasticity when levodopa is not being administered (46). LTP can be reversed in non-dyskinesia patients by administering levodopa (46). In LID patients, this therapeutic option may fail to reverse LTP (46). Deficiency of depotentiation exists in PD patients with LID (47). Theta and gamma wave patterns recorded by electroencephalography were found to be potent inducers of neuroplasticity (48). HF-rTMS was found to induce theta wave, and cTBS was found to induce theta and gamma in physiological conditions (49), which shows the capability of TMS to change neuroplasticity. Nevertheless, how rTMS-evoked neuroplasticity reverses dysfunctional neuroplasticity (that refers to lack of depotentiation) in the occurrence of LID and shows beneficial improvement on LID remains unknown.

### Neurotransmitter and Receptor Modulation

Imbalanced neurotransmitters are the major pathological mechanisms for LID. Studies have explored the roles of neurotransmitters and their receptors in TMS. Elevated GABA receptor levels have been found in postmortem samples of LID patients (50). The N-methyl-D-aspartate receptor antagonist, dextrorphan hydrochloride, has been shown to improve LID clinical outcomes (51). HF-rTMS increases the expression of amino acids (taurine, aspartate, and serine) and dopamine in the hypothalamic paraventricular nucleus and dorsal hippocampus, respectively, and decreases expression of arginine vasopressin in the hypothalamic paraventricular nucleus in healthy brains of rats and mice (52). LF-rTMS was, however, not capable of exhibiting any changes in neurotransmitters (53). Both LF-rTMS (54) and HF-rTMS (55) bring about an imbalance between glutamate and glutamine in healthy human brains. It has been shown that cTBS decreases vesicular glutamate transporters one and increases plasmatic glutamate transporters one in healthy rat brains (56). Upregulation of glutamate transporter and GABA transporter mRNAs have been reported in TMS-treated mice (57). Studies have confirmed the pivotal roles of glutamatergic and GABAergic neurotransmitters during TBS (58). More studies are needed to evaluate the role of neurotransmitters and receptors in LID patients during TMS therapy (59).

### Neurorestoration

Administration of glial cell line-derived neurotrophic factor (GDNF) improved LID both in patients and marmosets (60, 61). GDNF-mediated neurorestoration was revealed to selectively induce sprouting of dopaminergic cells without affecting GABAergic or serotonergic cells (62). In 50-sample animal research, rTMS alleviated LID with remarkably increased GDNF (63). However, cTBS alleviate LID with decreased brain-derived neurotrophic factor (BDNF) levels (18). Over-expression of BDNF was found to induce striatal serotonin fiber sprouting and lead to LID in 6-OHDA-lesioned rats (64). These findings suggest TMS might alleviate LID by sparing dopaminergic innervation and promoting serotonergic denervation. It is intriguing to note that different BDNF genotypes have a variable response to cTBS treatment (18). Val66Val carriers exhibited improvement of LID symptoms with decreased BDNF level after receiving cTBS treatment, while the Val66Met group showed no change for LID symptoms and the amount of BDNF as well (18).

### Blood Flow and Glucose Metabolism

Blood flow and glucose metabolism dissociation in subcortical regions, especially putamen, has been found implicated in the occurrence of LID (65, 66). Blood flow increased while glucose metabolism decreased in the putamen, be it in the medicated or unmedicated state (65, 66). Bilateral cerebellum cTBS alleviates LID by reducing [18F]-fluorodeoxyglucose positron emission tomography metabolism in bilateral cerebellar hemispheres and dentate nucleus (15). This study suggests that metabolic changes might mediate the efficacy of TMS (15). Till now, none studies have unraveled the relation of blood flow with TMS in the management of LID. Nevertheless, many studies indeed identified blood flow alteration after TMS in a wide range of brain regions and various diseases. Blood flow and glucose metabolism may imply some beneficial effects of TMS on LID.

## Prospects

To sum up, TMS has a neuromodulatory potential that might be successfully used in the clinical management of LID. However, more large randomized controlled studies of TMS application in LID are needed to understand better the underlying mechanisms, the efficacy evaluation, and optimization of stimulation protocols.

## Author Contributions

YW: writing—original draft preparation. X-bC: conceptualization. W-qZ, HZ, X-qZ, X-mY, CC, J-lW, and X-mY: resources. YX: writing—reviewing and editing. All authors contributed to the article and approved the submitted version.

## Funding

This work was supported by the National Key R&D Program of China (2017YFC1310300) and the National Natural Science Foundation of China (81873734, 81974200, and 81671108).

## Conflict of Interest

The authors declare that the research was conducted in the absence of any commercial or financial relationships that could be construed as a potential conflict of interest.

## Publisher's Note

All claims expressed in this article are solely those of the authors and do not necessarily represent those of their affiliated organizations, or those of the publisher, the editors and the reviewers. Any product that may be evaluated in this article, or claim that may be made by its manufacturer, is not guaranteed or endorsed by the publisher.

## Abbreviations

PD: Parkinson's disease
LID: levodopa-induced dyskinesia
TMS: transcranial magnetic stimulation
rTMS: repetitive TMS
HF-rTMS: high frequency rTMS
LF-rTMS: low frequency rTMS
TBS: theta burst stimulation
cTBS: continuous TBS
iTBS: intermittent TBS
MC: primary motor cortex
SMA: supplementary motor area
preSMA: pre-supplementary motor area
DLPFC: dorsolateral prefrontal cortex
IFC: inferior frontal cortex
SMA: supplementary motor area
SICI: short-interval intracortical inhibitions
LICI: long-latency intracortical inhibition
ICF: intracortical facilitation
SICF: short-interval intracortical facilitation
BDNF: brain-derived neurotrophic factor
GDNF: glial cell line-derived neurotrophic factor
LTP: long-term potentiation
MSO: maximum stimulator output
CFC: cross-frequency coupling
GABA: γ-Aminobutyric acid.

## References

[1] Tran TN Vo TNN Frei K Truong DD. Levodopa-induced dyskinesia: clinical features, incidence, and risk factors. J Neural Transm. (2018) 125:1109–117. 10.1007/s00702-018-1900-6 29971495

[2] Aradi SD Hauser RA. Medical management and prevention of motor complications in Parkinson's disease. Neurotherapeutics. (2020) 17:1339–65. 10.1007/s13311-020-00889-4 32761324 PMC7851275

[3] Dragasevic-Miskovic N Petrovic I Stankovic I Kostic VS. Chemical management of levodopa-induced dyskinesia in Parkinson's disease patients. Expert Opin Pharmacother. (2019) 20:219–30. 10.1080/14656566.2018.1543407 30411647

[4] Martini ML Mocco J Panov F. Neurosurgical approaches to levodopa-induced dyskinesia. World Neurosurg. (2019) 126:376–82. 10.1016/j.wneu.2019.03.056 30880213

[5] Koch G Brusa L Caltagirone C Peppe A Oliveri M Stanzione P. rTMS of supplementary motor area modulates therapy-induced dyskinesias in Parkinson disease. Neurology. (2005) 65:623–5. 10.1212/01.wnl.0000172861.36430.95 16116131

[6] Brusa L Versace V Koch G Iani C Stanzione P Bernardi G. Low frequency rTMS of the SMA transiently ameliorates peak-dose LID in Parkinson's disease. Clin Neurophysiol. (2006) 117:1917–21. 10.1016/j.clinph.2006.03.033 16887383

[7] Wagle-Shukla A Angel MJ Zadikoff C Enjati M Gunraj C Lang AE. Low-frequency repetitive transcranial magnetic stimulation for treatment of levodopa-induced dyskinesias. Neurology. (2007) 68:704–5. 10.1212/01.wnl.0000256036.20927.a5 17325284

[8] Filipovic SR Rothwell JC van de Warrenburg BP Bhatia K. Repetitive transcranial magnetic stimulation for levodopa-induced dyskinesias in Parkinson's disease. Mov Disord. (2009) 24:246–53. 10.1002/mds.22348 18951540

[9] Sayin S Cakmur R Yener GG Yaka E Ugurel B Uzunel F. Low-frequency repetitive transcranial magnetic stimulation for dyskinesia and motor performance in Parkinson's disease. J Clin Neurosci. (2014) 21:1373–6. 10.1016/j.jocn.2013.11.025 24631324

[10] Anja F Ann CA Sylvie DRA Veronique MA Sara S Annick VMA. Bilateral low frequency rTMS of the primary motor cortex may not be a suitable treatment for levodopa-induced dyskinesias in late stage Parkinson's disease. Parkinsonism Relat Disord. (2016) 22:54–61. 10.1016/j.parkreldis.2015.11.009 26777410

[11] Min Y Park JW Jin SU Jang KE Lee B Lee HJ. Neuromodulatory effects of offline low-frequency repetitive transcranial magnetic stimulation of the motor cortex: a functional magnetic resonance imaging study. Sci Rep. (2016) 6:36058. 10.1038/srep36058 27786301 PMC5081540

[12] Lohse A Meder D Nielsen S Lund AE Herz DM Løkkegaard A. Low-frequency transcranial stimulation of pre-supplementary motor area alleviates levodopa-induced dyskinesia in Parkinson's disease: a randomized cross-over trial. Brain Commun. (2020) 2:fcaa147. 10.1093/braincomms/fcaa147 33225277 PMC7667528

[13] Flamez A Wu G Wiels W Van Schuerbeek P De Mey J De Keyser J. Opposite effects of one session of 1 Hz rTMS on functional connectivity between pre-supplementary motor area and putamen depending on the dyskinesia state in Parkinson's disease. Clin Neurophysiol. (2021) 132:851–856. 10.1016/j.clinph.2020.12.024 33636601

[14] Koch G Brusa L Carrillo F Lo Gerfo E Torriero S Oliveri M. Cerebellar magnetic stimulation decreases levodopa-induced dyskinesias in Parkinson disease. Neurology. (2009) 73:113–9. 10.1212/WNL.0b013e3181ad5387 19597133

[15] Brusa L Ceravolo R Kiferle L Monteleone F Iani C Schillaci O. Metabolic changes induced by theta burst stimulation of the cerebellum in dyskinetic Parkinson's disease patients. Parkinsonism Relat Disord. (2012) 18:59–62. 10.1016/j.parkreldis.2011.08.019 21920793

[16] Cerasa A Koch G Donzuso G Mangone G Morelli M Brusa L. A network centred on the inferior frontal cortex is critically involved in levodopa-induced dyskinesias. Brain. (2015) 138:414–27. 10.1093/brain/awu329 25414038

[17] Ponzo V Picazio S Benussi A Di Lorenzo F Brusa L Caltagirone C. Altered inhibitory interaction among inferior frontal and motor cortex in l-dopa-induced dyskinesias. Mov Disord. (2016) 31:755–9. 10.1002/mds.26520 26861941

[18] Sanna A Follesa P Puligheddu M Cannas A Serra M Pisu MG. Cerebellar continuous theta burst stimulation reduces levodopa-induced dyskinesias and decreases serum BDNF levels. Neurosci Lett. (2020) 716:134653. 10.1016/j.neulet.2019.134653 31778767

[19] Rektorova I Sedlackova S Telecka S Hlubocky A Rektor I. Dorsolateral prefrontal cortex: a possible target for modulating dyskinesias in Parkinson's disease by repetitive transcranial magnetic stimulation. Int J Biomed Imaging. (2008) 2008:372125. 10.1155/2008/372125 18274665 PMC2233877

[20] Huang Y Edwards MJ Rounis E Bhatia KP Rothwell JC. Theta burst stimulation of the human motor cortex. Neuron. (2005) 45:201–6. 10.1016/j.neuron.2004.12.033 15664172

[21] Kujirai T Caramia MD Rothwell JC Day BL Thompson PD Ferbert A. Corticocortical inhibition in human motor cortex. The Journal of physiology. (1993) 471:501–19. 10.1113/jphysiol.1993.sp019912 8120818 PMC1143973

[22] Chen R Cros D Curra A Di Lazzaro V Lefaucheur J Magistris MR. The clinical diagnostic utility of transcranial magnetic stimulation: report of an IFCN committee. Clin Neurophysiol. (2008) 119:504–32. 10.1016/j.clinph.2007.10.014 18063409

[23] Barbin L Leux C Sauleau P Meyniel C Nguyen J Pereon Y. Non-homogeneous effect of levodopa on inhibitory circuits in Parkinson's disease and dyskinesia. Parkinsonism Relat D. (2013) 19:165–70. 10.1016/j.parkreldis.2012.08.012 23000298

[24] Pierantozzi M Palmieri MG Marciani MG Bernardi G Giacomini P Stanzione P. Effect of apomorphine on cortical inhibition in Parkinson's disease patients: a transcranial magnetic stimulation study. Exp Brain Res. (2001) 141:52–62. 10.1007/s002210100839 11685410

[25] Turjanski N Lees AJ. Gamma vinyl GABA in the treatment of levodopa-induced dyskinesias in Parkinson's disease. J Neurol Neurosurg Psychiatry. (1992) 55:413. 10.1136/jnnp.55.5.413 1602321 PMC489093

[26] Berardelli A Abbruzzese G Chen R Orth M Ridding MC Stinear C. Consensus paper on short-interval intracortical inhibition and other transcranial magnetic stimulation intracortical paradigms in movement disorders. Brain Stimul. (2008) 1:183–91. 10.1016/j.brs.2008.06.005 20633384

[27] Nakamura H Kitagawa H Kawaguchi Y Tsuji H. Intracortical facilitation and inhibition after transcranial magnetic stimulation in conscious humans. J Physiol. (1997) 498:817–23. 10.1113/jphysiol.1997.sp021905 9051592 PMC1159197

[28] Sanger TD Garg RR Chen R. Interactions between two different inhibitory systems in the human motor cortex. J Physiol. (2001) 530:307–17. 10.1111/j.1469-7793.2001.0307l.x 11208978 PMC2278414

[29] Ziemann U Tergau F Wassermann EM Wischer S Hildebrandt J Paulus W. Demonstration of facilitatory I wave interaction in the human motor cortex by paired transcranial magnetic stimulation. J Physiol. (1998) 511:181–90. 10.1111/j.1469-7793.1998.181bi.x 9679173 PMC2231091

[30] Guerra A Suppa A D'Onofrio V Di Stasio F Asci F Fabbrini G. Abnormal cortical facilitation and L-dopa-induced dyskinesia in Parkinson's disease. Brain Stimul. (2019) 12:1517–25. 10.1016/j.brs.2019.06.012 31217080

[31] Pascual-Leone A Valls-Solé J Wassermann EM Hallett M. Responses to rapid-rate transcranial magnetic stimulation of the human motor cortex. Brain. (1994) 117 (Pt 4):847–58. 10.1093/brain/117.4.847 7922470

[32] Chen R Classen J Gerloff C Celnik P Wassermann EM Hallett M. Depression of motor cortex excitability by low-frequency transcranial magnetic stimulation. Neurology. (1997) 48:1398–403. 10.1212/WNL.48.5.1398 9153480

[33] Ueno T Yamada J Nishijima H Arai A Migita K Baba M. Morphological and electrophysiological changes in intratelencephalic-type pyramidal neurons in the motor cortex of a rat model of levodopa-induced dyskinesia. Neurobiol Dis. (2014) 64:142–9. 10.1016/j.nbd.2013.12.014 24398173

[34] Cerasa A Messina D Pugliese P Morelli M Lanza P Salsone M. Increased prefrontal volume in PD with levodopa-induced dyskinesias: a voxel-based morphometry study. Movement Disord. (2011) 26:807–12. 10.1002/mds.23660 21384430

[35] Rascol O Sabatini U Brefel C Fabre N Rai S Senard JM. Cortical motor overactivation in parkinsonian patients with L-dopa-induced peak-dose dyskinesia. Brain. (1998) 121 (Pt 3):527–33. 10.1093/brain/121.3.527 9549528

[36] Cerasa A Pugliese P Messina D Morelli M Cecilia Gioia M Salsone M. Prefrontal alterations in Parkinson's disease with levodopa-induced dyskinesia during fMRI motor task. Movement Disord. (2012) 27:364–71. 10.1002/mds.24017 22076870

[37] Aron AR. Cortical and subcortical contributions to stop signal response inhibition: role of the subthalamic nucleus. J Neurosci. (2006) 26:2424–33. 10.1523/JNEUROSCI.4682-05.2006 16510720 PMC6793670

[38] Herz DM Haagensen BN Christensen MS Madsen KH Rowe JB Lokkegaard A. The acute brain response to levodopa heralds dyskinesias in Parkinson disease. Ann Neurol. (2014) 75:829–36. 10.1002/ana.24138 24889498 PMC4112717

[39] Ikeda A Yazawa S Kunieda T Ohara S Terada K Mikuni N. Cognitive motor control in human pre-supplementary motor area studied by subdural recording of discrimination/selection-related potentials. Brain. (1999) 122:915–31. 10.1093/brain/122.5.915 10355676

[40] Brooks DJ Piccini P Turjanski N Samuel M. Neuroimaging of dyskinesia. Ann Neurol. (2000) 47:S154–8; discussion S158–9. 10762143

[41] Lewis MM Du G Kidacki M Patel N Shaffer ML Mailman RB. Higher iron in the red nucleus marks Parkinson's dyskinesia. Neurobiol Aging. (2013) 34:1497–503. 10.1016/j.neurobiolaging.2012.10.025 23177595 PMC3570638

[42] Kishore A Popa T Balachandran A Chandran S Pradeep S Backer F. Cerebellar sensory processing alterations impact motor cortical plasticity in Parkinson's disease: clues from dyskinetic patients. Cereb Cortex. (2014) 24:2055–67. 10.1093/cercor/bht058 23535177

[43] Yoo HS Choi YH Chung SJ Lee YH Ye BS Sohn YH. Cerebellar connectivity in Parkinson's disease with levodopa-induced dyskinesia. Ann Clin Transl Neurol. (2019) 6:2251–60. 10.1002/acn3.50918 31643140 PMC6856615

[44] Belić JJ Halje P Richter U Petersson P Hellgren Kotaleski J. Untangling Cortico-striatal connectivity and cross-frequency coupling in L-DOPA-induced dyskinesia. Front Syst Neurosci. (2016) 10:26. 10.3389/fnsys.2016.00026 27065818 PMC4812105

[45] Noda Y Zomorrodi R Daskalakis ZJ Blumberger DM Nakamura M. Enhanced theta-gamma coupling associated with hippocampal volume increase following high-frequency left prefrontal repetitive transcranial magnetic stimulation in patients with major depression. Int J Psychophysiol. (2018) 133:169–74. 10.1016/j.ijpsycho.2018.07.004 30318052

[46] Morgante F Espay AJ Gunraj C Lang AE Chen R. Motor cortex plasticity in Parkinson's disease and levodopa-induced dyskinesias. Brain. (2006) 129:1059–69. 10.1093/brain/awl031 16476674

[47] Lago-Rodriguez A Ponzo V Jenkinson N Benitez-Rivero S Del-Olmo MF Hu M. Paradoxical facilitation after depotentiation protocol can precede dyskinesia onset in early Parkinson's disease. Exp Brain Res. (2016) 234:3659–67. 10.1007/s00221-016-4759-5 27566172

[48] Larson J Lynch G. Induction of synaptic potentiation in hippocampus by patterned stimulation involves two events. Science. (1986) 232:985–8. 10.1126/science.3704635 3704635

[49] Bergmann TO Karabanov A Hartwigsen G Thielscher A Siebner HR. Combining non-invasive transcranial brain stimulation with neuroimaging and electrophysiology: current approaches and future perspectives. Neuroimage. (2016) 140:4–19. 10.1016/j.neuroimage.2016.02.012 26883069

[50] Calon F Morissette M Rajput AH Hornykiewicz O Bedard PJ Di Paolo T. Changes of GABA receptors and dopamine turnover in the postmortem brains of parkinsonians with levodopa-induced motor complications. Mov Disord. (2003) 18:241–53. 10.1002/mds.10343 12621627

[51] Blanchet PJ Metman LV Mouradian MM Chase TN. Acute pharmacologic blockade of dyskinesias in Parkinson's disease. Mov Disord. (1996) 11:580–1. 10.1002/mds.870110516 8866502

[52] Keck ME Sillaber I Ebner K Welt T Toschi N Kaehler ST. Acute transcranial magnetic stimulation of frontal brain regions selectively modulates the release of vasopressin, biogenic amines and amino acids in the rat brain. Eur J Neurosci. (2000) 12:3713–20. 10.1046/j.1460-9568.2000.00243.x 11029641

[53] Poh EZ Hahne D Moretti J Harvey AR Clarke MW Rodger J. Simultaneous quantification of dopamine, serotonin, their metabolites and amino acids by LC-MS/MS in mouse brain following repetitive transcranial magnetic stimulation. Neurochem Int. (2019) 131:104546. 10.1016/j.neuint.2019.104546 31518601

[54] Tremblay S Beaulé V Proulx S de Beaumont L Marjańska M Doyon J. Relationship between transcranial magnetic stimulation measures of intracortical inhibition and spectroscopy measures of GABA and glutamate+glutamine. J Neurophysiol. (2013) 109:1343–9. 10.1152/jn.00704.2012 23221412 PMC3602833

[55] Michael N Gösling M Reutemann M Kersting A Heindel W Arolt V. Metabolic changes after repetitive transcranial magnetic stimulation (rTMS) of the left prefrontal cortex: a sham-controlled proton magnetic resonance spectroscopy (1 H MRS) study of healthy brain. Eur J Neurosci. (2003) 17:2462–8. 10.1046/j.1460-9568.2003.02683.x 12814378

[56] Mancic B Stevanovic I Ilic TV Djuric A Stojanovic I Milanovic S. Transcranial theta-burst stimulation alters GLT-1 and vGluT1 expression in rat cerebellar cortex. Neurochem Int. (2016) 100:120–7. 10.1016/j.neuint.2016.09.009 27623095

[57] Ikeda T Kobayashi S Morimoto C. Effects of repetitive transcranial magnetic stimulation on ER stress-related genes and glutamate, γ-aminobutyric acid and glycine transporter genes in mouse brain. Biochem Biophys Rep. (2019) 17:10–16. 10.1016/j.bbrep.2018.10.015 30456316 PMC6234257

[58] Li CT Huang YZ Bai YM Tsai SJ Su TP Cheng CM. Critical role of glutamatergic and GABAergic neurotransmission in the central mechanisms of theta-burst stimulation. Hum Brain Mapp. (2018) 40:2001–9. 10.1002/hbm.24485 30600571 PMC6865436

[59] Wei H Zhou Y Zhao J Zhan L. Risk factors and metabolism of different brain regions by positron emission tomography in Parkinson disease with disabling dyskinesia. Curr Neurovasc Res. (2019) 16:310–20. 10.2174/1567202616666191009102112 31622205

[60] Jenner P. Pathophysiology and biochemistry of dyskinesia: clues for the development of non-dopaminergic treatments. J Neurol. (2000) 247(Suppl. 2):II43–50. 10.1007/pl00007760 10991665

[61] Gill SS Patel NK Hotton GR O'Sullivan K McCarter R Bunnage M. Direct brain infusion of glial cell line-derived neurotrophic factor in Parkinson disease. Nat Med. (2003) 9:589–95. 10.1038/nm850 12669033

[62] Wu SS Frucht SJ. Treatment of Parkinson's disease: what's on the horizon? CNS Drugs. (2005) 19:723–43. 10.2165/00023210-200519090-00001 16142989

[63] Ba M Kong M Guan L Yi M Zhang H. Repetitive transcranial magnetic stimulation (rTMS) improves behavioral and biochemical deficits in levodopa-induced dyskinetic rats model. Oncotarget. (2016) 7:58802–812. 10.18632/oncotarget.11587 27613848 PMC5312277

[64] Tronci E Napolitano F Munoz A Fidalgo C Rossi F Bjorklund A. BDNF over-expression induces striatal serotonin fiber sprouting and increases the susceptibility to l-DOPA-induced dyskinesia in 6-OHDA-lesioned rats. Exp Neurol. (2017) 297:73–81. 10.1016/j.expneurol.2017.07.017 28757258

[65] Jourdain VA Tang CC Holtbernd F Dresel C Choi YY Ma Y. Flow-metabolism dissociation in the pathogenesis of levodopa-induced dyskinesia. JCI Insight. (2016) 1:e86615. 10.1172/jci.insight.86615 27699242 PMC5033758

[66] Aljuaid M Booth S Hobson DE Borys A Williams K Katako A. Blood flow and glucose metabolism dissociation in the putamen is predictive of levodopa induced dyskinesia in Parkinson's disease patients. Front Neurol. (2019) 10:1217. 10.3389/fneur.2019.01217 31824400 PMC6881455

